# Entrepreneurial Psychology and Motivation of Museum Cultural and Creative Product Development

**DOI:** 10.3389/fpsyg.2021.733943

**Published:** 2021-12-03

**Authors:** Ji Qi, Chaoli Song, Yan Wang

**Affiliations:** ^1^College of Cultural Industries Management, Communication University of China, Beijing, China; ^2^College of Literature and Law, Henan University of Animal Husbandry and Economy, Zhengzhou, China; ^3^School of Data Science and Intelligent Media, Communication University of China, Beijing, China

**Keywords:** museum, cultural and creative product development, entrepreneurial psychology, entrepreneurial motivation, cultural creativity and planning

## Abstract

More students who majored in design should be led to be self-employment, as they can use their major advantages to lead cultural and creative industries of museums toward a more creative direction. Based on the present situation of self-employment of college students related to cultural and creative industries, developing patterns and structure of cultural and creative industries of museums are analyzed to study the practical requirement of development of creative and cultural products of museums on the students majored in design. College students and graduates (within 2 years) in design major of a college in Zhejiang province who have experience in starting a business are invited to make the questionnaire survey and to study the practical problems with motives and barriers of starting a business. After the investigation of the entrepreneurial status of cultural and creative entrepreneurs of college students, it is found that the third year is the peak period for students to choose entrepreneurship, followed by the end of the senior year. Only 36.8% of respondents are satisfied with the results of entrepreneurship, which reflects that entrepreneurs do face many obstacles in the actual process of entrepreneurship, mainly due to a lack of experience and funds. The motivation for students to stick to entrepreneurial activities mainly focuses on “obtaining personal wealth” and “realizing self-worth.” In view of this situation, universities, society, and government can provide technical support and policy support to stimulate the entrepreneurial potential of cultural and creative entrepreneurs of college students, thus promoting the efficient development of museum cultural and creative industry.

## Introduction

In recent years, serious museums have been playing the “creative card” one after another, and they have repeatedly come up with new ideas in cultural and creative development. From the design of derivative products to the change of sales platform, static cultural relics are endowed with fashionable design. It turns museum “collections” into “commodities” and directly touches the consumer end in the form of “new retail,” turning cultural and creative development into a feasible project (Della Lucia and Trunfio, 2018). The cultural and creative products of the Alashan Museum in the Inner Mongolia autonomous region can be taken as an epitome of the development of cultural and creative industries of many local museums in China. According to public information, as of 2017, more than 2,500 museums, art galleries, and memorial halls in China have carried out cultural and creative development around their collections. Among them, the National Museum of China is one of the most typical representatives. It is not only widely recognized in the market and was put into market operation earlier, but it is also a characteristic in product development (Huang et al., 2014; Li and Ghirardi, 2019). In China, the cultural and creative industry development of the Palace Museum is undoubtedly the most successful case in domestic museums, and it is also the first to test the “Internet” (ShawHong, 2020). Capital has also begun to target this emerging market. Internet giants who have long coveted high-quality intellectual property (IP) resources of traditional culture have extended their tentacles to the cultural and museum industry after completing the IP layout of games, animation, film, and television industries (Pan et al., 2019).

The initial entrepreneurial process of entrepreneurs includes the acquisition of resources, the identification of entrepreneurial opportunities, the establishment of organizational structure, and the development of the market until the new enterprise was created successfully. In this process, it is the core of entrepreneurial opportunity identification. Entrepreneurs should transform valuable opportunities into the value creation of new enterprises. Entrepreneurial behavior refers to continuous entrepreneurial learning of the entrepreneur to realize entrepreneurship. Entrepreneurs use the mastered resources and skills for identifying and developing valuable entrepreneurial opportunities, and finally, complete the establishment of the enterprise. In the process of entrepreneurship, it is supposed to pay attention to the influence of characteristics and environmental uncertainty of entrepreneurs.

Based on the cultural and creative industry policy and innovative business model, what is first classified are the structure and development orientation of cultural and creative industry of the museum. Moreover, the questionnaire is used to analyze the current entrepreneurship situation of students majoring in design, including entrepreneurial motivation and entrepreneurial obstacles. Ultimately, constructive solutions are proposed according to the existing problems. Based on the present situation of starting a business of creative and cultural industries by college students, the research is expected to provide some reference and accordance to the development of creative and cultural industries of museums in China, which discusses how to make innovative development from the perspective of starting a business by college students. In the second section, what is reviewed is the related research on the entrepreneurial situation in the field of cultural and creative products and the entrepreneurial research of students majoring in design. In the third section, an introduction is made about the theoretical basis. Based on the development of museum cultural and creative products, analyzation is made on the innovation ability of entrepreneurs based on product development, and investigation is carried out on the entrepreneurial status of design and entrepreneurship students. In the fourth section, the results of empirical research and suggestions are provided to promote the entrepreneurial behavior of design students.

## Literature Review

Cultural and creative products are a kind of art derivatives, which are cultural and commemorative. At present, the development prospect of cultural and creative industry of the museum in China is still good since there is support from national policies to help in the development of cultural and creative products. With the adjustment and transformation of domestic industrial structure to the tertiary industry, design and service-related industries begin to attract more and more attention (Ch’ng et al., 2019). As the main driver of independent innovation, design plays a vital role in the promotion of the competitiveness of national pillar industries, which has become the key to the development of current design and cultural and creative industries. College students majoring in design usually have the spirit of innovation and creative thinking and can marketize the design results in practice. These factors make students majoring in design have unique entrepreneurial advantages (Jeong, 2018). However, in real life, due to the pressure of life, social support, and other factors, students majoring in design lack entrepreneurial knowledge, which leads to stagnation in the formulation of entrepreneurial goals and implementation of entrepreneurial steps. Therefore, it has become an urgent problem for them to use their own advantages to enhance their enthusiasm for innovation and entrepreneurship.

Bhansing et al. (2018) conducted research on the background of starting a business in the creative and cultural industries. The conclusion shows that with the engagement of an entrepreneur for the development of new products, the process of starting a business will become more stable for the creative and passionate personals. Landoni et al. (2020) pointed out that creative and cultural industries played an important role in the economic development of industrialized countries. But they are unmatured and easy to break up under specific limits or stressed situations, such as lack of ability of managing or the ability to develop complex value chains. Li et al. (2021) pointed out that perceiving innovation and experiencing value were two factors to promote the development of the creative and cultural industries of the museum. Thus, as to new entrepreneurs, they should highly regard the perception and experience of consumers to design their products.

## Research Methodology

### Connotation and Influencing Factors of the Entrepreneurial Behavior of Enterprises

Human behavior is an important research object of management, and the research results of behavior science greatly enrich the management theory. For the research of behavior, important work is to decompose the behavior, analyze the behavior process, reveal the reasons that affect the behavior, and identify the behavior rules. Opportunity is the core element of entrepreneurship, and entrepreneurship cannot do without opportunity. Opportunity identification is the key behavior in entrepreneurial activities. In many entrepreneurial stories, opportunity identification seems to be completed in an instant. Opportunities do not always exist. If it does exist, entrepreneurs need to act quickly. Thus, speed has become a key factor for entrepreneurial success.

Entrepreneurial behavior is the process of opportunity development of new ventures. The subject of entrepreneurship is the entrepreneur or entrepreneurial team. From the micro level, the subject of entrepreneurial behavior is the entrepreneur individual or group, entrepreneurial behavior is carried out around the entrepreneurial thinking of entrepreneur individual, and the new entrepreneurial behavior is that of the entrepreneur individual. The initial entrepreneurial behavior is characterized by the outflow of resources, which is a kind of value-consuming activity. The consumption of value will bring greater value, whereas the result of entrepreneurial behavior is the creation of value after the enterprise is successfully created.

The whole process of entrepreneurial activity starts from the entrepreneurial intention, searching and discovering opportunities, then decides to set up new enterprises, and finally develops entrepreneurial opportunities. The key point lies in opportunity identification and discovery. Only by finding useful entrepreneurial opportunities can entrepreneurs start a business and complete entrepreneurial behavior. In the whole process of starting a business, entrepreneurs should continue looking for and developing entrepreneurial opportunities, so that enterprises can really grow up. From the perspective of cognitive theory, it is found that the identification process of entrepreneurial opportunities will affect the development process of entrepreneurial opportunities, and then affect entrepreneurial behavior. Hence, a systematic and planned search for entrepreneurial opportunities will make it easier for entrepreneurs to find entrepreneurial opportunities and carry out entrepreneurial activities more actively.

### The Structure and Development Model of Cultural and Creative Industries in Museums

**FIGURE 1:**
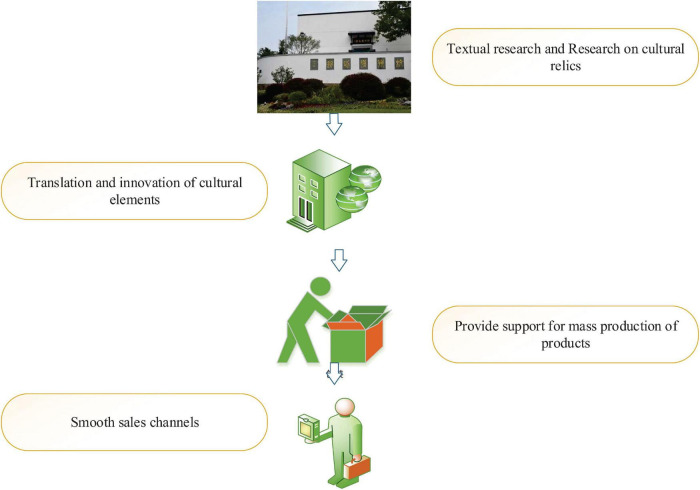
Operation process of museum cultural and creative industry chain.

United Nations Educational, Scientific, and Cultural Organization defines cultural industry as an industry with cultural creation, commodity production, and profit making. Cultural and creative industries are booming with the competitive value of “soft power” among countries (Heng, 2010). Among them, museums have rich cultural value and economic value, which is an important factor to measure the cultural and creative level of a country. Its cultural and creative industry chain consists of museums, design companies, manufacturers, and sellers. Figure 1 shows the operation process of each other. Museums have all kinds of cultural resources IP, whose role is to research and investigate cultural relics, and they are the “leaders” in the development of museum cultural and creative products; design companies are good at modern translation, innovation, and transformation of cultural elements, and in the formation of production drawings; manufacturers have advanced manufacturing technology to provide support for mass production of products; sellers have smooth sales channels, first-hand consumer demand, and rich marketing experience. If the above four parties can work together, they will achieve internal win-win results and promote the healthy development of the cultural and creative industry of museums, which is the “ideal mode” to lead them to sustainable development (Sutton et al., 2017).

Through the efforts of museum practitioners, many museums’ cultural and creative products emerge with creativity, culture, aesthetics, and practicality. Cultural and creative products provide museums with excellent added value and they become the extension of brand value. However, compared with the development of museum cultural industry in advanced countries in the world, China’s museum cultural and creative industry is still in the initial exploration and cultivation stage, and there are still problems, such as insufficient product creativity, lack of production technology, and imperfect market operation mechanism (Yang and Černevičiūtė, 2017). Under the background of cultural tourism integration, the museum industry should continue to innovate and explore a new development mode of the cultural and creative industry.

First, museums should play their own role and give full play to their resource advantages. For example, they should choose the collection with good looks to interpret the connotation of the cultural relics, such as modeling, structure, and decoration, and excavate the historical stories behind the cultural relics. Based on the interest points of the audience, the historical story should be narrated in a humorous or sensational style without changing the history, to provide a comprehensive and rich collection of information for designers (McAndrew et al., 2020). Furthermore, museums should change their ideas and actively integrate into the cultural and creative market. Based on special collections of the museum, they should integrate resources from all walks of life and carry out a variety of art licensing attempts, to fully connect IP resources of the museum with social industry resources. Only in this way, the development mode of high efficiency, high quality, and low cost can be obtained, the win-win connotation can be realized, and the museum cultural and creative industry chain can be improved (Gray et al., 2015). Then, it can make cultural and creative industry of the museum form a creative, cooperative, and marketing cultural and creative development mode. Specifically, the museum can authorize well-known high-quality manufacturers in tourism, food, stationery, home furnishing, and other industries. Usually, these manufacturers have excellent design teams and mature marketing plans, and their financial system is huge, and so they can develop cultural and creative products with market potential. Museums only need to check the historical and cultural connotation elements of creative products. Manufacturers can provide a continuous stream of high-quality cultural and creative products for the museum cultural and creative industry, and solve the problems of product storage and after-sales. Moreover, the cultural and creative design needs to meet the needs of collection culture, practical life, scientific, and technological innovation. The museum can cooperate with high-tech product companies to develop creative products with the “Internet+” elements (Cong et al., 2019). They can license the appropriate IP resources to high-tech companies, reflect the characteristics of cultural relics from product modeling and packaging design, and make cultural and creative products of museum enter the public from cultural relics. Meanwhile, they need to pay attention to the practicality of products and close to people’s lives, so those cultural and creative products can keep the historical temperature, and solve practical needs of people for goods. Finally, museums can combine local characteristic IP with museum elements to develop a series of cultural and creative products. Local characteristics belong to the category of regional culture, which includes the natural environment, urban landscape, customs, and so on, and highlights a unique way of life and ideas of people. Developing a series of cultural and creative products can meet the commemorative and cultural needs of tourism services and help museums carry out social education.

### Development Mode and Typical Cases of Museum Cultural and Creative Products

Compared with ordinary commodities, museum cultural and creative products also have a cultural function, which is a kind of consumer goods with the function of conveying opinions and lifestyle. Cultural and creative products ultimately need to enter the market, and so they need to be embedded with three characteristic elements of cultural connotation, industrial activities, and popular orientation (Kourtit and Nijkamp, 2019). Cultural and creative products are the specific form of cultural transformation into the cultural industry. They should not only inherit the history and culture but also integrate the popular factors with unique creativity to obtain economic benefits. As an industrial activity, successful cultural and creative products can make continuous profits for the industry, whereas cultural and creative products that lack innovation and tend to copy will be separated from the artistry of products and cannot promote the development of the industry. The combination of culture and the popular elements of the times can make the current popular culture more recognized by the audience (Camarero et al., 2019). Designers must further understand the representation of culture, make the detailed culture resonate with the specific audience in the design process, and promote the creative products with traditional and popular culture.

**FIGURE 2:**
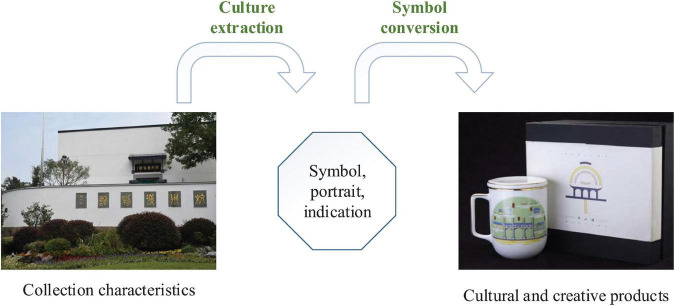
Design transformation process of cultural and creative products. Images reproduced with permission from Suzhou Museum.

Through the production of cultural and creative products, cultural and creative industries regard it as the carrier of cultural connotation, traditional spirit, and other symbols. Figure 2 shows the design transformation process of cultural and creative products. There is a non-single line relationship among cultural and creative industries, cultural and creative products, and consumer behavior. Cultural and creative products need to convey symbolic value through the media system so that they have a greater chance to promote the actual consumption behavior (Marchegiani, 2018).

Nowadays, the emergence of many public museums makes them compete with each other. Joint product development with enterprises is a new way for museums to attract tourists. At present, most museums in China lack professional designers, and so museums usually choose to cooperate with independent enterprises to design products (Anoegrajekti et al., 2018; Comunian and England, 2019). For example, Nanjing Museum, as a national first-class museum, has a special cultural and creative department, but there is no corresponding designer. However, in 2012, Nanjing Museum adopted the cooperative development mode. Designers will design creatively based on the cultural connotation of the cultural relics, and “communicate” with consumers to develop cultural and creative products that satisfy consumers. While industrial development is improved, cultural resources can be inherited.

The development of museum cultural and creative products needs to be focused on five elements: culture, innovation, brand, publicity, and education. Table 1 shows the details. The marketing objects of museum cultural and creative products mainly include special exhibition crowd, fans, and tourists. Different audiences have different cognition and self-interpretation of traditional culture, which will be reflected in the consumer market (Belfiore, 2020). Designers can know the preferences and needs of different consumer groups according to the feedback of product sales, which can be used as a reference for the next cultural and creative product development. The cultural and creative products that link traditional culture with modern technology should shoulder the mission of cultural continuity and propaganda, and further enhance the influence and popularity of the museum.

**TABLE 1:** Five elements of museum cultural and creative product development.

Characteristic elements	Specific requirement
Culture	Cultural and creative products come from culture. Different types of museums should design unique cultural and creative products according to their own collections and cultural resources.
Innovation	Innovative thinking should run through the whole process of cultural and creative product development to avoid the situation that the form and style of museum cultural and creative products tend to be consistent.
Brand	Cultural and creative products are the basis of building museum brand effect, which is mainly reflected in the brand symbol of the museum marked on the series of products.
Publicity	The sale of cultural and creative products is a way for museums to publicize themselves. Through product publicity, consumers can have a deeper understanding of the collections.
Education	Cultural and creative products should not only be limited to visiting cultural relics in museums but also permeate the historical heritage of cultural relics in museums.

**FIGURE 3:**
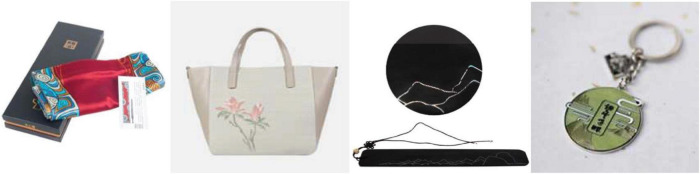
Four series of cultural and creative products of Suzhou Museum. Images reproduced with permission from Suzhou Museum.

The development of cultural and creative products in the Suzhou Museum is taken as an example. Suzhou Museum attaches great importance to and vigorously develops the cultural industry. Now, it has formed a comprehensive development mode with the art store in the museum as the core, and the audience lounge, wisteria Bookstore counter, and Taobao online store are the auxiliary. The growing sales of the Suzhou Museum of cultural and creative products are inseparable from its unique style in the field of cultural and creative development. Its development is committed to exploring cultural elements and highlights from the cultural relics in its collection, integrating them with modern and contemporary handicrafts, and creating representative Suzhou crafts. In 2009, the State Administration for Industry and Commerce approved the trademark of “Suzhou Museum.” The trademark is established in the cultural products of the Suzhou Museum. At present, the cultural and creative products of Suzhou Museum are developed under the four series of categories: treasure of the town museum, Wumen four families, Suzhou Museum Architecture, and smoke and cloud passing by, as shown in Figure 3. Cultural and creative products cover food, jewelry, stationery, bags, arts and crafts, and other fields, with obvious Suzhou style. In the online store of Suzhou Museum, 96 pieces of cultural and creative products are currently on sale, including seven categories. Table 2 illustrates the specific contents.

**TABLE 2:** Cultural and creative products sold online in Suzhou Museum.

Categories	Products
Stationery	notebook, hand account, tape, bookmark, folder, post-card, mobile phone case
Home furnishing	decorative painting
Clothes and accessories	backpacks, silk scarves
General merchandise	luggage tag, key chain, cup, refrigerator sticker
Food	ST color porcelain lotus bowl shape cookies, jasmine wine, creative tea bag
Jewelry and ornaments	Bracelets, earrings, brooches
Publication	3D book

Consumers generally have a good evaluation of the cultural and creative products of Suzhou Museum, believing that the design is ingenious and the product quality is up to standard. However, from the design point of view, most of the products lack creativity. Some of the products are copies of successful cultural and creative products all over the world, which is not conducive to the long-term development of cultural promotion and the cultural and creative industry. In addition, the most important factors affecting consumers to buy products are the cultural value and cost performance, and so more attention should be paid to it in future product design and development.

### An Analysis of the Main Body Innovation System of Chinese Cultural and Creative Industries

Since the 11th Five-Year Plan period, China has regarded the ability of independent innovation as the main breakthrough point of economic development. The strategic goal of the “innovative country” proposed by China is the accurate grasp of the new situation by the government. Under this policy, the ministry of culture attaches great importance to the cultural and creative industry because of its unique innovation characteristics, which shows its importance to the development of the national economy.

The colleges and universities of China have been shouldering the important task of personnel training, training lots of cultural and creative industry talents for the society, and undertaking the historical task of improving the artistic cultivation and aesthetic level of “cultural and creative consumers” in the whole society. In the process of promoting the development of cultural and creative enterprises, colleges and universities play an important role. As to actively cultivate the brand of cultural and creative enterprises, they give full play to their own resource advantages and provide relatively favorable development conditions and space. The exchange and cooperation of scientific research and technology between universities and cultural and creative enterprises are also strengthened. Through the joint establishment of cultural and creative research institutions, cultural and creative enterprises provide a broad platform for the development of scientific research practice for experts and scholars (He and Huang, 2018; Shih, 2018; Stejskal and Hajek, 2019). Colleges and universities fully utilize their own advantages in human resources and actively carry out relevant scientific research for cultural and creative enterprises. The research results, as an important basis for the decision-making of cultural and creative enterprises, play an important guiding role.

Government research institution plays an important role in the technological innovation of cultural and creative industries. Government research institutions have a certain government background, can play the government’s advantage of resource concentration, and are superior to universities in a research environment, personnel, equipment, capital investment, and other aspects. The government research institutions can get more financial support and policy guidance, which ensures the feasibility and advanced nature of technology research in the cultural and creative industries. Meanwhile, it plays a positive leading role in the promotion of research results, which lays a solid foundation for the formation of the industrial chain of cultural and creative industries (Feng and Chen, 2020).

### Entrepreneurial Forms of Students Majoring in Design in Cultural and Creative Industries

To meet the needs of self-development, college students can use their existing resources and professional ability to realize their self-worth in the economic environment. At the national level, China strongly encourages and supports entrepreneurship of college students, and eagerly expects and needs a group of college -students entrepreneurs to provide innovative products and services for the progress of the country and society (Liu and Chen, 2021; Tunio et al., 2021). Besides, a series of preferential policies have been introduced to help the steady growth of entrepreneurial enterprises of college students, including enterprise registration, tax incentives, social security subsidies, capital docking, entrepreneurship training, and many other aspects. However, for college students, entrepreneurship has a higher risk than employment, and employment activities are bound to bring stable remuneration and income to the employees. However, entrepreneurs can obtain certain benefits through entrepreneurial activities, and may also bear economic losses due to failure. The core talents of the museum cultural and creative industry discussed are college students majoring in art and design (Kratzer et al., 2017). Although the cultural creative industry of China starts late and the degree of industrialization is relatively low, the development situation cannot be underestimated. Therefore, it is of great practical significance to analyze the employment and entrepreneurial intention of college students majoring in related majors, which can provide a reference for the policy-making of museum cultural and creative industry development.

Based on the group characteristics of outstanding ability and team cooperation of college students, as well as the characteristics of cultural and creative industries, such as high knowledge and high added value, the entrepreneurial ways of college students in cultural and creative industries are mainly divided into three forms: group entrepreneurship, concept entrepreneurship, and part-time entrepreneurship. As the cultural and creative industries require high professional skills for entrepreneurs, it takes a long time for individuals to learn and practice to fully master the skills required by the business. Therefore, group entrepreneurship is the most common form of entrepreneurship for college students who majored in design (Durana et al., 2019; Teixeira et al., 2019). Team members master their own professional skills and are responsible for a specific business, which can effectively make up for their own skill defects, and complete the corresponding business with streamlined and high quality. Concept entrepreneurship is a way to attract investors and obtain capital, talents, and other resources by virtue of entrepreneurial ideas. The entrepreneurship of cultural and creative industries needs entrepreneurs to have some experience in the innovation of projects and social contacts. For college student entrepreneurs, they usually only have innovative ideas and projects. Through the concept of entrepreneurship, they can complete the fund-raising and talent absorption in a short time and can put their entrepreneurial ideas into practice more smoothly. Parttime entrepreneurship has certain advantages for the cultural and creative industry, mainly because compared with traditional industries, the cultural and creative industry is more flexible in time arrangement, and has more applications of computer technology and the Internet (Chen et al., 2018; Gundolf et al., 2018). Therefore, some college students of cultural and creative industry design entrepreneurs will choose the form of carrying out the employment of related enterprises and independent entrepreneurship at the same time.

## Experimental Design and Results

### Current Situation of Entrepreneurship of College Students Majoring in Design

The cultural and creative entrepreneurs of college students in Zhejiang Province are selected as the research object. A questionnaire is used to understand the entrepreneurial motivation, entrepreneurial barriers, and other practical problems of college students. Furthermore, constructive solutions are put forward to improve the entrepreneurial ability of college students. A total of 190 questionnaires are distributed and 185 valid questionnaires are collected. The specific content of the questionnaire is presented in the Appendix. According to statistics, the proportion of male and female students participating in the questionnaire is about 2:1; there are 49 registered companies, 43 individual businesses, 20 studios, 30 online stores, and 43 others.

### Investigation on the Current Situation of Independent Entrepreneurship of Students in Product Design Majors

**FIGURE 4:**
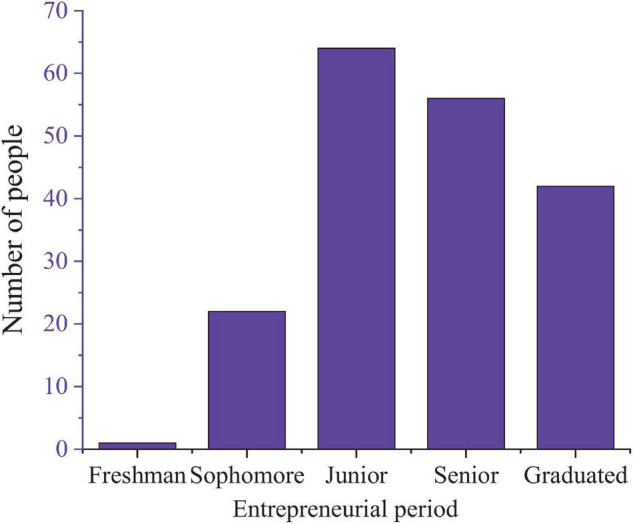
Entrepreneurial time choice of college students.

First, it summarizes the overall situation of cultural and creative entrepreneurs’ entrepreneur of college students, including the choice of entrepreneurial time and the identification of the entrepreneurial team. The survey shows that the third year is the peak period for students to choose entrepreneurship, with 64 students, accounting for 34.6%, followed by the end of the fourth year, accounting for 22.7%. Figure 4 shows the specific results. This is related to the relatively free course time and rich entrepreneurial knowledge reserve of students in the graduation stage. In entrepreneurial groups, 67.6% of respondents act as project sponsors, which also shows that college students are more active and innovative than other social groups in entrepreneurial activities.

According to the survey, most entrepreneurial firms of college students are small and medium-sized enterprises, among which enterprises with one to five employees account for 66.5%, whereas enterprises with more than 20 employees only account for 9.7%. In this case, due to the lack of capital accumulation and less social experience, it is more reliable for college students to start their own businesses in small and medium-sized enterprises. The business types and coverage of large enterprises are more comprehensive, and college-student entrepreneurs need to accumulate more to improve the quality of enterprises and expand the scale of enterprises.

**FIGURE 5:**
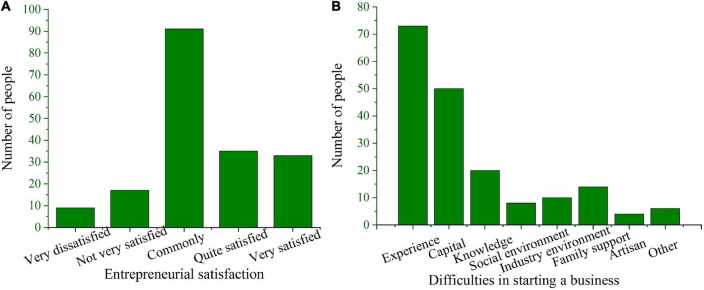
Entrepreneurial satisfaction and main difficulties of college students. **(A)** Entrepreneurial satisfaction and **(B)** difficulties in starting a business.

To further understand the current situation of entrepreneurship of college students, entrepreneurial satisfaction and the most important entrepreneurial difficulties of college students are investigated. Figure 5A is the result. It reveals that about half of the entrepreneurs hold a neutral attitude toward the overall satisfaction of entrepreneurship, and only 36.8% felt “relatively satisfied” or “very satisfied” with the entrepreneurial results. This also reflects that college student entrepreneurs do face a lot of obstacles in the actual entrepreneurial process, resulting in overall low entrepreneurial satisfaction. The main difficulties faced by college students in the process of entrepreneurship are investigated, and Figure 5B presents the result. It reveals that lack of experience and lack of funds are still the main difficulties faced by entrepreneurs. Bhansing et al. (2018) investigated the entrepreneurial motivation of design entrepreneurs. This specific type of entrepreneurial motivation is mainly reflected in the transformation of creativity into cultural and creative products. This is consistent with the main results of entrepreneurial motivation of design majors discussed in this study.

### Analysis of Entrepreneurship Psychology and Motivation of Cultural and Creative Entrepreneurs of College Students

At present, cultural and creative industries are developing rapidly. For cultural and creative entrepreneurs of college students, their entrepreneurial motivation has a certain impact on the sustainability of entrepreneurial behavior. The entrepreneurial motivation of college students in this major is investigated through eight questions. Table 3 shows the topics and survey results. Among them, 1–5, respectively, means “totally disagree,” “disagree,” “general,” “relatively agree,” and “very agree.”

**TABLE 3:** Entrepreneurial motivation of college students majoring in product design.

Questions	1	2	3	4	5
because of hobbies	14	16	59	40	56
in order to realize self-worth	10	12	38	48	77
in order to challenge yourself	21	27	55	37	45
in order to obtain personal wealth	10	15	22	63	78
the employment situation is grim; in order to solve the employment problem	37	33	53	33	29
like the free working atmosphere	12	18	43	44	68
influenced by entrepreneurship policy	29	33	55	28	40
create more jobs for the society	39	30	57	37	22

From the perspective of entrepreneurial motivation, entrepreneurial reasons of the respondents mainly focus on the three reasons of “obtaining personal wealth,” “realizing self-worth,” and “liking free working atmosphere,” which also shows that for the new generation of college students, self-development and self-experience are the main motivation to choose to start a business. Relatively speaking, there are fewer students who choose to start a business for the sake of social effect or job supplement, and the publicity of the society and universities should also be strengthened in this aspect. Chen et al. (2017) used motivation and entrepreneurial theory to understand the willingness of entrepreneurs to start and launch entrepreneurship and found that the motivation of entrepreneurs is mainly realized by meeting basic financial needs, work-life balance needs, social reputation needs, and career achievement needs. What is supplemented here is the entrepreneurial motivation of design majors. Personal development and self-experience are the two most important motivations of entrepreneurship for college students.

### Summary of an Entrepreneurial Model of Achievement Transformation Practice

In the era of the rise of museum cultural and creative industries, the energy released by cultural and creative industries will bring opportunities and power to college students entrepreneurs. Therefore, for students in design majors, it is a feasible scheme to start their own business with the mode of “achievement transformation practice,” which can be divided into the following specific measures (Table 4).

**TABLE 4:** Entrepreneurial model of achievement transformation practice.

Entrepreneurial model	Specific content
entering business incubator to start a business	through the preparation of product business plan, application is made to enter the business incubator, to obtain technical support and site support for product development
cooperating with enterprises to start a business through the cooperation platform	through cooperation with enterprises with production capacity, innovative technology is transformed into products, and enterprise management and organization methods are learned.
guiding entrepreneurship through design competition	Design competitions usually have business operation background, which can bring potential design products to the market or seek venture investors.
studio business model	Studio business model has a high degree of flexibility, feasibility and low cost, and students can use the technical support environment provided by the school to start a business.
building a network platform to start a business	In the form of network intermediary, the relationship between “manufacturer” and “consumer” is established to provide services for both sides.

As the manifestation of technology, design plays a guiding role in technology. For students in design majors, in the process of entrepreneurial practice and commercialization of design results, the realization of different products needs different technical support. In addition to the technology provided by cooperative enterprises, teachers and students of design majors in colleges and universities can also provide technical support for creative design. As an individual entrepreneur, a student can build a bridge between teachers and students through the auxiliary departments of colleges and universities, gather other students with technical ability and entrepreneurial ideas, let the students who are starting a business find partners who can cooperate, and encourage potential entrepreneurs to have the courage to start a business.

## Conclusion

Entrepreneurship is an activity with high market competition pressure and a high elimination rate. For the cultural and creative industries of museums, which have sprung up in recent years, with the help of cultural and creative products, museum culture can be brought into reality and historical details can be integrated into the lives of people. The promotion of cultural and creative products is inseparable from creative design. Creative design thinking is the foundation of building an art museum. To guide more students majoring in design to engage in independent entrepreneurship and contribute to the development of museum cultural and creative industry, the entrepreneurship status of product design by students is investigated.

Once entrepreneurs have the idea of entrepreneurship, they should first obtain the knowledge, skills, and other resources needed for entrepreneurship, and use the resources to find entrepreneurial opportunities in the market. Those with entrepreneurial intention aim to have entrepreneurial behavior, so they will spontaneously collect the information needed to achieve the goal of starting a business. The entrepreneurial motivation of college students is mainly to “obtain personal wealth” and “realize self-worth”; lack of experience and lack of funds are still the main difficulties faced by college student entrepreneurs. Colleges and universities can provide students with technical guidance and entrepreneurship education and hold entrepreneurship experience exchange meetings.

New methods are given through the research to motivate the college students who majored in design to broaden their practice of starting a business. Society can provide students who have business ideas with platforms to cooperate with enterprises, through which students can store experience and resources for self-employment. Besides the talent of design majors, the creative and cultural industries of museums need talents of majors in management, operation, propaganda, and so on. Measures of absorbing related talents can be studied in later researches. An investigation is made on the specific group of design majors in the self-employment of college students to analyze the entrepreneurial conditions they enjoy, the entrepreneurial characteristics they have, and the innovative practice mode they can carry out. In future research, if the current situation of the entrepreneurial practice of students is subdivided from different design majors to find out the best implementation method of innovative practice assumption, the innovative practice assumption will be more in line with the requirements of entrepreneurs and the market. The research and exploration results are of reference and guidance for the specific entrepreneurial activities of design students.

## Data Availability Statement

The raw data supporting the conclusions of this article will be made available by the authors, without undue reservation.

## Ethics Statement

The studies involving human participants were reviewed and approved by Communication University of China Ethics Committee. The patients/participants provided their written informed consent to participate in this study. Written informed consent was obtained from the individual(s) for the publication of any potentially identifiable images or data included in this article.

## Author Contributions

All authors listed have made a substantial, direct, and intellectual contribution to the work, and approved it for publication.

## Conflict of Interest

The authors declare that the research was conducted in the absence of any commercial or financial relationships that could be construed as a potential conflict of interest.

## Publisher’s Note

All claims expressed in this article are solely those of the authors and do not necessarily represent those of their affiliated organizations, or those of the publisher, the editors and the reviewers. Any product that may be evaluated in this article, or claim that may be made by its manufacturer, is not guaranteed or endorsed by the publisher.

## Appendix

### College Students’ Entrepreneurship Questionnaire

Dear students:

Hello! We are carrying out the research of college students’ entrepreneurship, and this questionnaire survey is a part of the research. The purpose of the survey is to study the current college students’ understanding of entrepreneurial activities, entrepreneurial environment, entrepreneurial knowledge, innovation and entrepreneurship education, please help fill in. The survey is anonymous and the information you provide is used only for research purposes and will never be disclosed to any third party. Hope you provide a real idea of each problem. There is no right or wrong answer to the question. Please type “√” or fill in the appropriate content on the options that are in line with your actual situation or ideas. Thank you for your participation and support in this study!

1. Which stage are you in during your first venture:

A. First; B. Second; C. Third; D. Fourth.

2. Your identity in the entrepreneurial team:

A. project leader (initiator); B. participant.

3. Why do you want to start a business? (Please score 1–5 for “totally disagree,” “disagree,” “generally,” “relatively agree,” and “very agree,” respectively).

(1) For hobbies

(2) To achieve self-worth

(3) To challenge yourself

(4) To gain personal wealth

(5) To address employment issues under the severe employment situation

(6) Free working atmosphere

(7) Affected by entrepreneurial policies

(8) Creating more jobs for society

4. The current scale (quantity of employees) of your enterprise:

A. 1–5; B. 6–10; C. 11–20; D. 21–50; E. >50.

5. Overall satisfaction with your current entrepreneurial status:

A. very unsatisfied; B. unsatisfied; C. general; D. satisfied; E. very satisfied.

6. The main difficulties of your entrepreneurship at present:

A. lacks entrepreneurial experience and connections; B. lacks entrepreneurial capital;

C. lacks entrepreneurial knowledge and skills; D. lacks good psychological quality;

E. social entrepreneurial environment is not ideal; F. industry environment is not ideal;

G. lacks family support; H. entrepreneurial team instability; I. lacks core technical staff; J. other.

7. What entrepreneurial support and services schools and society provide:

A. entrepreneurship funding support;

B. entrepreneurship policy support (e.g., drop-out entrepreneurship, credit substitution, etc.);

C. entrepreneurship mentor to guide; D. entrepreneurship Site to support;

E. hardware support; F. help start-ups promote;

G. entrepreneurial atmosphere; H. none.

8. Which entrepreneurial related practice activities you have participated in:

A. Entrepreneurship Competition; B. Entrepreneurship Roadshow; C. Entrepreneurship Successful Person Report Exchange; D. Entrepreneurial Salon and Symposium; E. Entrepreneurial Training Camp Activities; F. Visit Mature Enterprises or Entrepreneurial Parks; G. founded a simulation company; H. internship in the employing unit; I. served as student cadre; J. none.
